# Fees for Professional Services

**Published:** 1860-01

**Authors:** 


### "FEES FOR PROFESSIONAL SERVICES."

We noticed in "The North American Medico-Chirurgical Review" an excellent editorial on the above, and as it holds forth the views which we have always entertained, we cannot do better than make some reference to it, as the same subject has also engaged the attention of some of our profession; and, again, because considerable fault has been found for the last ten years with us by many in the profession for not rating our charges higher. We never have engaged in the discussion of this subject, nor do we expect to; but to answer all anxious inquirers, we will set forth our views for the purpose of explaining how we have been governed, and how we expect to be, unless the law of the land interpose to direct us, and then, doubtless, we will be found on the right side. We believe with the writer of the article referred to, that the "regular fee-bills, regulating the charges of ordinary practice; a custom which we have always regarded as absurd and unjust, since it places, in this particular, all practitioners, whatever may be their respective merits, upon the same level; whereas every man should be permitted to charge according to his skill and the nature of his services, not forgetting the circumstances of his patients." If patients complain among each other about the difference between the charges of different dentists, it has nothing to do with the subject between dentists. We would not be willing to be governed in our practice by what any dentist should consider a fair equivalent for *his own services*, or what any patient should think was sufficient, whether it was more or less than we, and our patients who preferred us, chose to agree upon. We have always said, when complaint has been made, that if we can not operate for a patient *at all*, and he is obliged to pay another, whom he places confidence in, three times as much, it is none of our business; the patient has no right to cite us as a standard, or bring another down to our charges, no matter how low they are.

If old dentists choose to limit their practice and make high charges, and those whom they have served long and well can afford to employ them at such rates, and who know their services are valuable and successful, and they make that known to their patients before the operations are performed, no one has any cause to complain; nor does any other dentist, young or old, lower the dignity of his profession by operating for a smaller fee. But we do not hold to the doctrine that a dentist who has raised value of his operations in his own estimation, or rates his charges justly above the mass, or his usual rates with which his patients are familiar, has a right to make his claim what he pleases after the operations are finished; a patient has a right to judge whether he can pay an increase of charge or not. We know that there has been a great deal of complaint by patients on this part of the subject, and justly too; and instead of the dentists raising the dignity of the profession by it, they do a great deal to lower it. No one can hope to raise the dignity of his profession by any unreasonable things; but he has a right to bargain for whatever he pleases, providing his patient is a party to the bargain. We have many patients in our practice, some who left their former dentists years ago, on account of high charges, and they pay us more at present than they paid the dentists whom they left.

It is simply impossible for one patient to pay as much as another, the community through, for dental services—and we regard it as a morbid idea for a young man, or even an old one, to be all the time lamenting that he is not receiving as much as another dentist or an older one. We are quite sure that it has never given us any trouble, that older or younger men in the profession receive more for their services than we. We know very well that an intelligent patient of ours paid fifteen dollars to a dentist for plugging a tooth which we had not time to do; while we had never charged more than five dollars for similar operations; and neither of us lost any reputation. The more free the matter of charge is left between the dentist and the community, without the restrictions of fee-bills, the better it will be for all parties. Charges should always be regulated in every dentist's practice according to the community he lives in, so as to be pleasant and agreeable on both sides. If not, the war between them will keep him in trouble all the time. If there is no other and nobler feeling holding together a professional man and his practice than pecuniary interest, it is a very cold and unworthy connection.

We received a letter a short time since from a respectable member of the profession, in our city, on the subject of charges for our operations in plugging, which we did not answer, as we had long since intended to make an article on the subject, which would answer his case—as it was not new to us, and many would be answered instead of one. He remarked that many of his new patients had complained of his charges being higher than ours; that we had operated for their relatives and friends for two dollars a plug; but he had contradicted it—and was he right?—as he wished to charge, although young in the profession, so as not to lower its dignity. We will answer here that he was not right in contradicting it; we doubtless operate for relatives and friends of the patients of many dentists for two dollars, and always expect to do so as long as we have fingers and health to work; we operate for many such, and we do not intend to cut them off. Many of them can not afford to pay more; but, doubtless, many can; but they have struggled upward through life like ourselves, with small means, as patients of ours and as friends, and they shall stay with us so long as God gives us life, if it is their pleasure so to do, no matter what rate of charge we shall make from time to time upon the mass. Nor do we believe that they should be taken as a rule to govern others by—either patients or dentists—nor will we ever interpose between patients and dentists, to regulate their charge. —*Dental Cosmos.*

